# First records of *Selaginellakraussiana* and *Selaginellasubcordata* from Senegal (Selaginellaceae)

**DOI:** 10.3897/BDJ.12.e134350

**Published:** 2024-12-03

**Authors:** Paterne A. B. Mingou, Mathieu Gueye, Theophile Bayet, Christophe Cambier

**Affiliations:** 1 Laboratoire de Botanique, IFAN Ch. A. Diop, BP 206, Dakar, Senegal Laboratoire de Botanique, IFAN Ch. A. Diop, BP 206 Dakar Senegal; 2 Laboratoire de Botanique, IRL 3189, Environnement, Santé et Société, IFAN Ch. A. Diop BP 206, Dakar, Senegal Laboratoire de Botanique, IRL 3189, Environnement, Santé et Société, IFAN Ch. A. Diop BP 206 Dakar Senegal; 3 Sorbonne Université, IRD, UCAD,UGB UMI UMMISCO, F-75006, Paris, France Sorbonne Université, IRD, UCAD,UGB UMI UMMISCO, F-75006 Paris France

**Keywords:** Pteridophytes, Kédougou, Ziguinchor, distribution, ecology, herbaria

## Abstract

**Background:**

The monogeneric family Selaginellaceae is made up of about 700 species distributed throughout the world, but the most concentrated part is in tropical and subtropical areas. According to the most recent infrageneric classification of the genus *Selaginella*, six or seven subgenera can be recognised and perhaps 700 species. The genus is monophyletic, cosmopolitan, characterised by the presence of rhizophores, ligulated leaves and has a reniform adaxial sporangia with two type of spores (heterospory).

**New information:**

The records of two species are reported, that is *S.kraussiana* (Kunze) A.Braun and *S.subcordata* A.Braun ex Kuhn, which are new for the state of Senegal. Ecological traits, especially related to the habitat and altitude-elevation distribution, are also described for these species. Both species were collected in the south of Senegal, more precisely in the region of Kédougou for *S.Kraussiana* and in the regions of Kédougou, Tambacounda and Ziguinchor for *S.subcordata.*

## Introduction

Lycophyta is a monophyletic group of plants with microphylls and adaxial reniform sporangia (Webster 1992, Banks et al. 2011, Wan et al. 2023). The lycophyte genus *Selaginella* alone constitutes the family Selaginellaceae and the order Selaginellales following the phylogenetic classification of the Pteridophyte Phylogeny Group (PPG I) (PPG 2016). This genus is generally characterised by dorsiventral leaves of different sizes with four rows, with a ligule at the base of each leaf and sporophyll as well, the presence of rhizophores and heterospory (Webster 1992, Thomas 1997, Weststrand and Korall 2016a, Weststrand and Korall 2016b, Valdespino 2020). The genus has a cosmopolitan distribution and comprises around 700-800 species (Stefanović and Rakotondrainibe 1996, Banks 2009, Zhou and Zhang 2015, PPG 2016, Valdespino 2020). Species in the genus have adapted to an impressive range of habitats, including temperate forests (alpine and arctic habitats), tropical rainforest and exhibit drought tolerance (Banks 2009, Valdespino 2020).

In Senegal, there were just a few studies focused on Pteridophytes, i.e. Mingou and Gueye (2017), Mingou et al. (2021) and Mingou et al. (2023). However, collected material during studies on the flora of Senegal was the subject of two types of flora (Berhaut 1954, Berhaut 1967). This made it possible to establish estimates of this taxonomic group based on bibliographic data (Ba and Noba 2001, Roux 2009). These authors reported three species of *Selaginella* (*S.myosurus* (Sw.) Alston, *S.versicolor* Spring and *S.tenerrima* A.Braun ex Kuhn) in Senegal. Despite these few publications on the Pteridophytes of Senegal, the pteridoflora remains poorly studied, although it occupies a significant place in the Senegalese flora (Berhaut 1954, Berhaut 1967, Ba and Noba 2001). Therefore, we have been conducting research work on this group throughout Senegal for several years. The present work reports the presence of two species of *Selaginella* newly listed for the Senegalese Pteridological flora during our explorations. Their habitats and geographic distributions are also described.

## Materials and methods

Several prospecting surveys were carried out in rice fields, palm groves, water ponds, forests and gallery forests, mountains and waterfalls on various administrative regions of Senegal while considering the specificity of environments as well. Fertile samples were collected, pressed and preserved at the IFAN Herbarium, which is the oldest botanical collection in French-speaking Africa. However, when mature individuals were not available, sterile samples were collected. For each sample, duplicates or even more samples were collected, depending on the size of the population encountered. Wanting to facilitate the identification and confirmation of these species, photos of the plant and its organs before and after sampling were joined with the herbarium collections. A fragment of the blade was also taken every time and preserved in silica gel according to the protocol of Gaudeul and Rouhan (2013) for subsequent molecular studies. For this purpose, a herbarium specimen was always accompanied by photos of the entire plant before collection, a silica gel sample under the same number with the specimen and a note on its ecology.

At the same time, herbarium specimens of Pteridophytes preserved in the BR, DAKAR, IFAN and P Herbaria were checked (herbaria acronyms according to Thiers (2024)). Indeed, BR and P were selected for their centuries-old specimens and with current collaboration and exchanges with Senegalese Herbaria. P is the World Reference Herbarium which contains one of the largest collections of African specimens, particularly from West Africa, including numerous type specimens (Le Bras et al. 2017). All the Pteridophyte material from Senegal in these four herbaria were analysed. Thus, an inventory of the collections of pteridophytes was realised from Senegal in these herbaria, taking care to note all the information appearing on the labels. For certain specimens preserved at P and BR, whose manipulation was not possible, digital consultation of their images was adopted (https://science.mnhn.fr/institution/mnhn/item/search and https://www.botanicalcollections.be/#/fr/search/specimen). In Senegal, the RIHA database of the IFAN Ch. A. Diop (IFAN) herbarium was consulted to extract the specimens of Senegal pteridophytes, which were preserved there. As the DAKAR herbarium does not have a database, we extracted all the pteridophyte specimens from Senegal from the boxes in order to note all the information on the labels.

Initial species identification in the field was later verified and validated in the laboratory by making a comparison with referenced specimens from DAKAR and IFAN herbaria. IPNI (https://www.ipni.org) allowed us to update the names of the species according to their correct names. At the higher rank (genus and family), we took into account the recent phylogenetic works by Smith et al. (2006), Schuettpelz et al. (2007) and Christenhusz et al. (2011), but the adopted classification is that of PPG (2016).

For the description of the species, we used a LEICA M80 binocular stereomicroscope equipped with an IC80 HD type camera connected to a computer. Images taken with the LAS EZ 4 software made it possible to describe and compare the morphology of the specimens with the specimens available in the databases of JSTOR (https://plants.jstor.org/) and African Pteridophytes (https://www.fernsofafrica.com/index.php). Our collections and occurrence data obtained from the Global Biodiversity Information Facility (GBIF; http://www.gbif.org/occurrence/search?taxon) have enabled the production of mapping species distribution in Africa.

## Data resources

A total of 1160 herbarium specimens of Pteridophytes (665 new and 495 old collections), preserved in the IFAN, DAKAR, BR and P herbaria, were consulted, including 43 of the genus *Selaginella*. As a result, the Senegalese pteridoflora is enriched with two species (*Selaginellakraussiana* and *Selaginellasubcordata*) that had not been previously reported in the country. They come from our field collections for *Selaginellakraussiana* and cumulatively from historical collections and our field collections for *Selaginellasubcordata*. For the latter, the old collections are all stocked in BR.

## Taxon treatments

### Selaginella kraussiana

(Kunze) A.Braun, 1859

01F8E561-30A9-5162-BC7C-D01A1C5E8B27

urn:lsid:ipni.org:names:90781-3

#### Materials

- **Type status:** Other material. **Occurrence:** catalogNumber: IFAN63162; recordNumber: PABM0261; recordedBy: Mingou Paterne A.B. et al; occurrenceID: 8BE90795-7568-5157-B6FB-9DB3D9B06C36; **Taxon:** taxonID: urn:lsid:ipni.org:names:90781-3; scientificName: Selaginellakraussiana; scientificNameAuthorship: (Kunze) A.Braun; **Location:** country: Senegal; stateProvince: Kédougou; locality: Southern part of the Dindefélo waterfall cliff, at 100 m high; verbatimElevation: 391 m; locationRemarks: label transliteration: "Kédougou, Dindefélo, 2019.09.23, Mingou Paterne A.B. et al."; [Kédougou Dindefélo 391 m, 12°21'54.3"N 012°19'30.1"W, 2019.09.23, Lithophyte, Mingou Paterne A.B. et al.]; verbatimCoordinates: 12°21'54.3"N 012°19'30.1"W; decimalLatitude: 12.3651; decimalLongitude: -12.325; georeferenceProtocol: GPS; **Identification:** identifiedBy: Mingou Paterne A.B.; dateIdentified: 2020; **Event:** samplingProtocol: climbing; eventDate: 2019.09.23; habitat: Lithophyte; **Record Level:** language: fr; institutionCode: IFAN; collectionCode: Pteridophytes; ownerInstitutionCode: IFAN; basisOfRecord: PreservedSpecimen
- **Type status:** Other material. **Occurrence:** catalogNumber: IFAN63163; recordNumber: PABM0266; recordedBy: Mingou Paterne A.B. et al; occurrenceID: E1B7830E-7A33-5DA6-932D-4E6456B596E6; **Taxon:** taxonID: urn:lsid:ipni.org:names:90781-3; scientificName: Selaginellakraussiana; scientificNameAuthorship: (Kunze) A.Braun; **Location:** country: Senegal; stateProvince: Kédougou; locality: Southern part of the Dindefélo waterfall cliff, at 90 m high; verbatimElevation: 385 m; locationRemarks: label transliteration: "Kédougou, Dindefélo, 2019.09.23, Mingou Paterne A.B. et al."; [Kédougou Dindefélo 385 m, 12°21'54.0"N 012°19'29.7"W, 2019.09.23, Lithophyte, Mingou Paterne A.B. et al.]; verbatimCoordinates: 12°21'54.0"N 012°19'29.7"W; decimalLatitude: 12.365; decimalLongitude: -12.3249; georeferenceProtocol: GPS; **Identification:** identifiedBy: Mingou Paterne A.B.; dateIdentified: 2020; **Event:** samplingProtocol: climbing; eventDate: 2019.09.23; habitat: Lithophyte; **Record Level:** language: fr; institutionCode: IFAN; collectionCode: Pteridophytes; ownerInstitutionCode: IFAN; basisOfRecord: PreservedSpecimen
- **Type status:** Other material. **Occurrence:** catalogNumber: IFAN63164; recordNumber: PABM0271; recordedBy: Mingou Paterne A.B. et al; occurrenceID: C326944D-2F29-5C38-A17C-684EDBECE1E9; **Taxon:** taxonID: urn:lsid:ipni.org:names:90781-3; scientificName: Selaginellakraussiana; scientificNameAuthorship: (Kunze) A.Braun; **Location:** country: Senegal; stateProvince: Kédougou; locality: Southern part of the Dindefélo waterfall cliff, at 80 m high; verbatimElevation: 376 m; locationRemarks: label transliteration: "Kédougou, Dindefélo, 2019.09.23, Mingou Paterne A.B. et al."; [Kédougou Dindefélo 376 m, 12°21'53.8"N 012°19'29.2"W, 2019.09.23, Lithophyte, Mingou Paterne A.B. et al.]; verbatimCoordinates: 12°21'53.8"N 012°19'29.2"W; decimalLatitude: 12.3649; decimalLongitude: -12.3248; georeferenceProtocol: GPS; **Identification:** identifiedBy: Mingou Paterne A.B.; dateIdentified: 2020; **Event:** samplingProtocol: climbing; eventDate: 2019.09.23; habitat: Lithophyte; **Record Level:** language: fr; institutionCode: IFAN; collectionCode: Pteridophytes; ownerInstitutionCode: IFAN; basisOfRecord: PreservedSpecimen
- **Type status:** Other material. **Occurrence:** catalogNumber: IFAN63165; recordNumber: PABM0273; recordedBy: Mingou Paterne A.B. et al; occurrenceID: D9F0DF26-92CC-56DB-8D64-0A1AD53A220F; **Taxon:** taxonID: urn:lsid:ipni.org:names:90781-3; scientificName: Selaginellakraussiana; scientificNameAuthorship: (Kunze) A.Braun; **Location:** country: Senegal; stateProvince: Kédougou; locality: Northern part of the Dindefélo waterfall cliff, at 50 m high; verbatimElevation: 376 m; locationRemarks: label transliteration: "Kédougou, Dindefélo, 2019.09.23, Mingou Paterne A.B. et al."; [Kédougou Dindefélo 376 m, 12°21'53.8"N 012°19'29.0"W, 2019.09.23, Lithophyte, Mingou Paterne A.B. et al.]; verbatimCoordinates: 12°21'53.8"N 012°19'29.0"W; decimalLatitude: 12.365; decimalLongitude: -12.3247; georeferenceProtocol: GPS; **Identification:** identifiedBy: Mingou Paterne A.B.; dateIdentified: 2020; **Event:** samplingProtocol: climbing; eventDate: 2019.09.23; habitat: Lithophyte; **Record Level:** language: fr; institutionCode: IFAN; collectionCode: Pteridophytes; ownerInstitutionCode: IFAN; basisOfRecord: PreservedSpecimen
- **Type status:** Other material. **Occurrence:** catalogNumber: IFAN63166; recordNumber: PABM0275; recordedBy: Mingou Paterne A.B. et al; occurrenceID: 0CBDC4BD-4521-5116-87AA-E0E21AEEA6DD; **Taxon:** taxonID: urn:lsid:ipni.org:names:90781-3; scientificName: Selaginellakraussiana; scientificNameAuthorship: (Kunze) A.Braun; **Location:** country: Senegal; stateProvince: Kédougou; locality: Southern part of the Dindefélo waterfall cliff, at 60 m high; verbatimElevation: 369 m; locationRemarks: label transliteration: "Kédougou, Dindefélo, 2019.09.23, Mingou Paterne A.B. et al."; [Kédougou Dindefélo 369 m, 12°21'52.0"N 012°19'25.6"W, 2019.09.23, Lithophyte, Mingou Paterne A.B. et al.]; verbatimCoordinates: 12°21'52.0"N 012°19'25.6"W; decimalLatitude: 12.3644; decimalLongitude: -12.3238; georeferenceProtocol: GPS; **Identification:** identifiedBy: Mingou Paterne A.B.; dateIdentified: 2020; **Event:** samplingProtocol: climbing; eventDate: 2019.09.23; habitat: Lithophyte; **Record Level:** language: fr; institutionCode: IFAN; collectionCode: Pteridophytes; ownerInstitutionCode: IFAN; basisOfRecord: PreservedSpecimen
- **Type status:** Other material. **Occurrence:** catalogNumber: IFAN63167; recordNumber: PABM0283; recordedBy: Mingou Paterne A.B. et al; occurrenceID: 486C572D-BCE0-5E5F-B9DE-89C68AB069F2; **Taxon:** taxonID: urn:lsid:ipni.org:names:90781-3; scientificName: Selaginellakraussiana; scientificNameAuthorship: (Kunze) A.Braun; **Location:** country: Senegal; stateProvince: Kédougou; locality: Southern part of the Dindefélo waterfall cliff, at 50 m high; verbatimElevation: 358 m; locationRemarks: label transliteration: "Kédougou, Dindefélo, 2019.09.23, Mingou Paterne A.B. et al."; [Kédougou Dindefélo 358 m, 12°21'53.4"N 012°19'27.7"W, 2019.09.23, Lithophyte, Mingou Paterne A.B. et al.]; verbatimCoordinates: 12°21'53.4"N 012°19'27.7"W; decimalLatitude: 12.3648; decimalLongitude: -12.3244; georeferenceProtocol: GPS; **Identification:** identifiedBy: Mingou Paterne A.B.; dateIdentified: 2020; **Event:** samplingProtocol: climbing; eventDate: 2019.09.23; habitat: Lithophyte; **Record Level:** language: fr; institutionCode: IFAN; collectionCode: Pteridophytes; ownerInstitutionCode: IFAN; basisOfRecord: PreservedSpecimen
- **Type status:** Other material. **Occurrence:** catalogNumber: IFAN63168; recordNumber: PABM0289; recordedBy: Mingou Paterne A.B. et al; occurrenceID: 1A3C271E-CBB5-5AED-B64F-15E6A0C046F7; **Taxon:** taxonID: urn:lsid:ipni.org:names:90781-3; scientificName: Selaginellakraussiana; scientificNameAuthorship: (Kunze) A.Braun; **Location:** country: Senegal; stateProvince: Kédougou; locality: Northern part of the Dindefélo waterfall cliff, at 30 m high; verbatimElevation: 327 m; locationRemarks: label transliteration: "Kédougou, Dindefélo, 2019.09.23, Mingou Paterne A.B. et al."; [Kédougou Dindefélo 327 m, 12°21'52.1"N 012°19'26.7"W, 2019.09.23, Lithophyte, Mingou Paterne A.B. et al.]; verbatimCoordinates: 12°21'52.1"N 012°19'26.7"W; decimalLatitude: 12.3645; decimalLongitude: -12.3241; georeferenceProtocol: GPS; **Identification:** identifiedBy: Mingou Paterne A.B.; dateIdentified: 2020; **Event:** samplingProtocol: climbing; eventDate: 2019.09.23; habitat: Lithophyte; **Record Level:** language: fr; institutionCode: IFAN; collectionCode: Pteridophytes; ownerInstitutionCode: IFAN; basisOfRecord: PreservedSpecimen
- **Type status:** Other material. **Occurrence:** catalogNumber: IFAN63169; recordNumber: PABM0297; recordedBy: Mingou Paterne A.B. et al; occurrenceID: 248922BF-8ADD-5C17-B656-67136C49C522; **Taxon:** taxonID: urn:lsid:ipni.org:names:90781-3; scientificName: Selaginellakraussiana; scientificNameAuthorship: (Kunze) A.Braun; **Location:** country: Senegal; stateProvince: Kédougou; locality: Southern part of the Dindefélo waterfall cliff, at 20 m high; verbatimElevation: 318 m; locationRemarks: label transliteration: "Kédougou, Dindefélo, 2019.09.23, Mingou Paterne A.B. et al."; [Kédougou Dindefélo 318 m, 12°21'53.2"N 012°19'25.5"W, 2019.09.23, Lithophyte, Mingou Paterne A.B. et al.]; verbatimCoordinates: 12°21'53.2"N 012°19'25.5"W; georeferenceProtocol: GPS; **Identification:** identifiedBy: Mingou Paterne A.B.; dateIdentified: 2020; **Event:** samplingProtocol: climbing; eventDate: 2019.09.23; habitat: Lithophyte; **Record Level:** language: fr; institutionCode: IFAN; collectionCode: Pteridophytes; ownerInstitutionCode: IFAN; basisOfRecord: PreservedSpecimen
- **Type status:** Other material. **Occurrence:** catalogNumber: IFAN63170; recordNumber: PABM0305; recordedBy: Mingou Paterne A.B. et al; occurrenceID: 0F3BF39C-D97C-57B5-B757-0B5CEF8C205F; **Taxon:** taxonID: urn:lsid:ipni.org:names:90781-3; scientificName: Selaginellakraussiana; scientificNameAuthorship: (Kunze) A.Braun; **Location:** country: Senegal; stateProvince: Kédougou; locality: Southern part of the Dindefélo waterfall cliff, at 10 m high; verbatimElevation: 308 m; locationRemarks: label transliteration: "Kédougou, Dindefélo, 2019.09.23, Mingou Paterne A.B. et al."; [Kédougou Dindefélo 308 m, 12°21'52.9"N 012°19'27.3"W, 2019.09.23, Lithophyte, Mingou Paterne A.B. et al.]; verbatimCoordinates: 12°21'52.9"N 012°19'27.3"W; georeferenceProtocol: GPS; **Identification:** identifiedBy: Mingou Paterne A.B.; dateIdentified: 2020; **Event:** samplingProtocol: climbing; eventDate: 2019.09.23; habitat: Lithophyte; **Record Level:** language: fr; institutionCode: IFAN; collectionCode: Pteridophytes; ownerInstitutionCode: IFAN; basisOfRecord: PreservedSpecimen
- **Type status:** Other material. **Occurrence:** catalogNumber: IFAN63171; recordNumber: PABM0369; recordedBy: Mingou Paterne A.B. et al; occurrenceID: F744B4C6-8A78-5A6B-B952-6D9DBAA1252C; **Taxon:** taxonID: urn:lsid:ipni.org:names:90781-3; scientificName: Selaginellakraussiana; scientificNameAuthorship: (Kunze) A.Braun; **Location:** country: Senegal; stateProvince: Kédougou; locality: Watercourse on the Bembou-Massa axis, towards Sabadola; verbatimElevation: 133 m; locationRemarks: label transliteration: "Kédougou, Bembou-Massa, 2019.09.29, Mingou Paterne A.B. et al."; [Kédougou Bembou-Massa 133 m, 12°51'26.2"N 011°53'14.6"W, 2019.09.29, Lithophyte, Mingou Paterne A.B. et al.]; verbatimCoordinates: 12°51'26.2"N 011°53'14.6"W; decimalLatitude: 12.8573; decimalLongitude: -11.8874; georeferenceProtocol: GPS; **Identification:** identifiedBy: Mingou Paterne A.B.; dateIdentified: 2020; **Event:** samplingProtocol: sweeping; eventDate: 2019.09.29; habitat: Lithophyte; **Record Level:** language: fr; institutionCode: IFAN; collectionCode: Pteridophytes; ownerInstitutionCode: IFAN; basisOfRecord: PreservedSpecimen

#### Description

**Figure 1.**
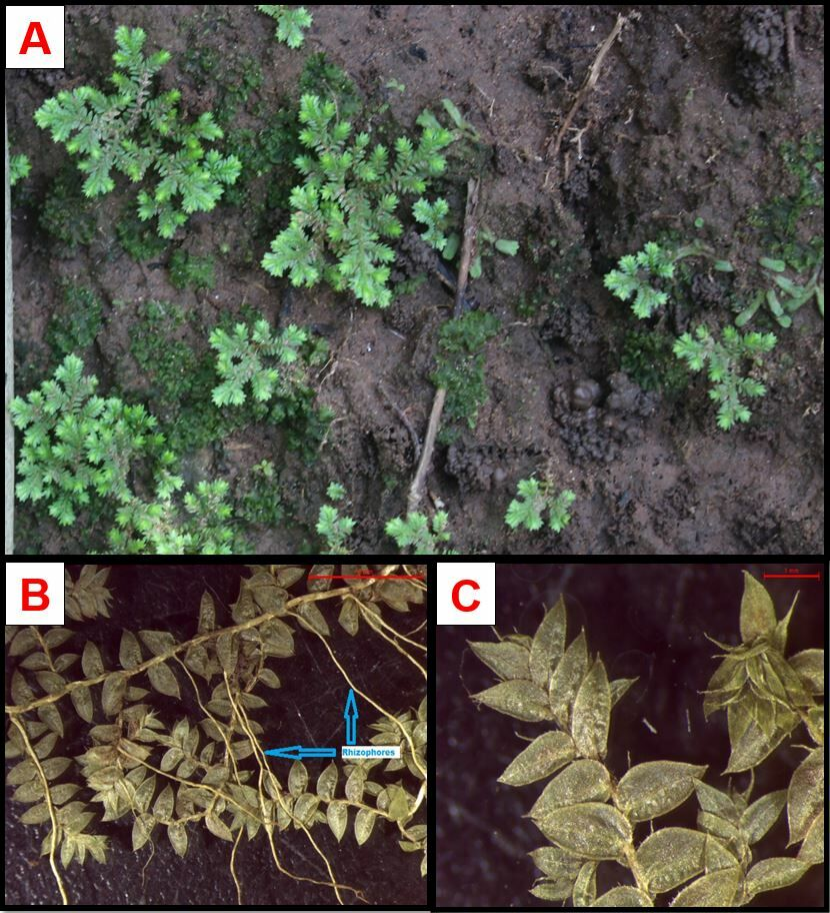
*Selaginellakraussiana* (Kunze) A.Braun. **A** Plant in its environment; **B** Arrangement of rhizophores throughout the stem; **C** Arrangement of leaves at the top of the branch.

Plant terrestrial (Fig. 1A) mainly lithophytic, diffuse mat-forming. Stems widely creeping, dichotomous, flat, articulated, glabrous. Rhizophores on the upper side of the stems along the entire length of the stem, 1-3 mm in diameter (Fig. 1B). Leaves dimorphic, delicate; lateral leaves almost perpendicular to the stem, green, well-spaced, lanceolate, 2-3 × 0.3-1.1 mm, base rounded, margin slightly transparent to green, toothed, apex pointed (Fig. 1C); median leaves lanceolate to linear-lanceolate, 2-2.5 × 0.2-0.7 mm, apex acuminate, base cordate and unequal, margins finely toothed. Strobili, solitary, with only one basal megasporangium, situated on short side branches, 2-25 mm; sporophylls toothed, strongly tapering towards the apex, base glabrous, marge denticulate, apex acuminate; megasporophylls larger than microsporophylls, in groups of four, lanceolate-elliptical to falcate-lanceolate; microsporophylls lanceolate to narrowly ovate-lanceolate.

#### Distribution

**Figure 2.**
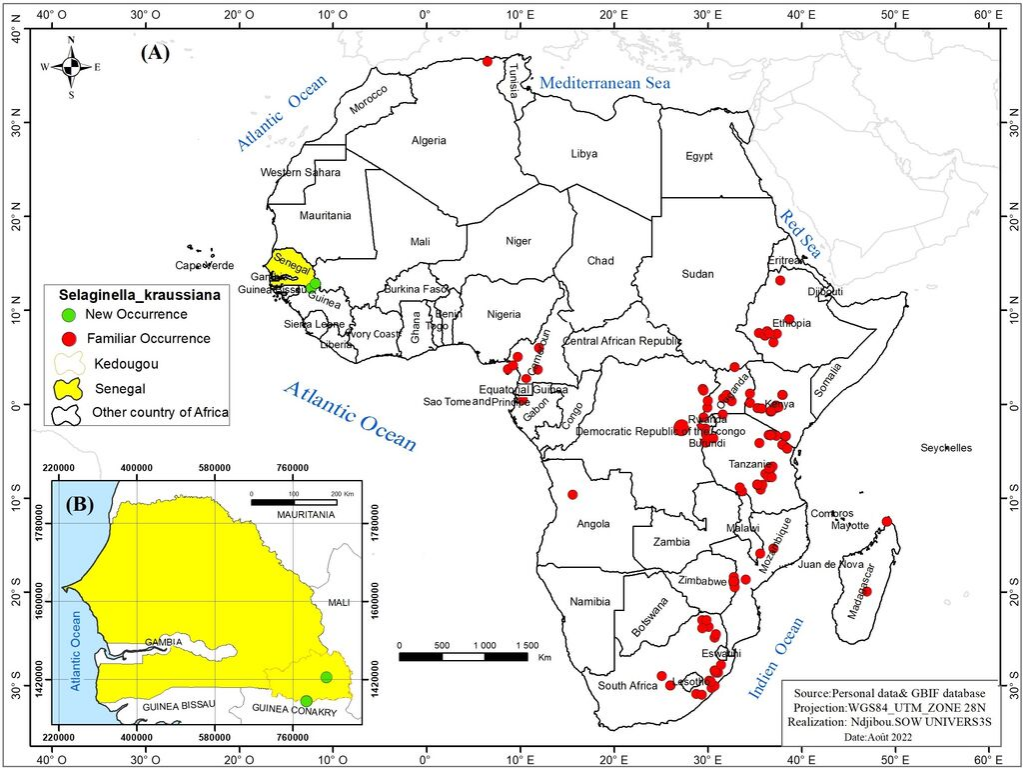
Distribution map *Selaginellakraussiana* (Kunze) A.Braun in Africa **(A)** and Senegal **(B).**

Distribution in Senegal: Kédougou Region (Fig. 2B).

Distribution in Africa: Algeria, Angola, Burundi, Cameroon, Comoros, Ethiopia, Kenya, Madagascar, Malawi, Mozambique, Uganda, DRC, Rwanda, Sao Tome and Principe, Sudan, South Africa, Tanzania and Zimbabwe (GBIF.org 2022a) (Fig. 2A).

#### Ecology

*Selaginellakraussiana* is found predominantly as a lithophyte at altitudes between 133 and 391 m a.s.l., on both sides of the Dindefélo Falls cliff, but it is more confined on the south side at almost all levels of the cliff at 100 m, 90 m, 80 m, 60 m, 50 m, 20 m and 10 m high and on the north side at 50 m and 30 m. The souhern part of the cliff is humid and very sunny, with few species of trees, unlike the northern part which is less humid and very sunny. On the Dindefélo cliff, *S.kraussiana* was found each time under plant cover in association with pteridophyte species such as *Aleutopterisfarinosa* (Forssk.) Fée, *Blotiellacurrorii* (Hook.) R.M. Tryon, *Dicranopterislinearis* (Burm.f.) Underw. and *Selaginellaversicolor* Spring, but also with byophytes and spermatophytes such as *Dombeyaquinqueseta* (Delie) Excell, *Mitragynainermis* (Willd.) K.Schum. and *Raphiasudanica* A.Chev. It forms a green carpet on the rock. It is also found on land on the banks of a river on the Bembou-Massa axis.

### Selaginella subcordata

A.Braun ex Kuhn, 1868

B1FC593C-E1E5-5D25-905B-9DD1C1E807B4

urn:lsid:ipni.org:names:77186660-1

#### Materials

- **Type status:** Other material. **Occurrence:** catalogNumber: IFAN63172; recordNumber: PABM0150; recordedBy: Mingou Paterne A.B. et al; occurrenceID: 4EDB192B-BF75-574B-AFBB-74734EF7A5F2; **Taxon:** taxonID: urn:lsid:ipni.org:names:77186660-1; scientificName: Selaginellasubcordata; scientificNameAuthorship: A.Braun ex Kuhn; **Location:** country: Senegal; stateProvince: Kédougou; locality: Sédou stream in Ebarack, not far from the Bridge; verbatimElevation: 116 m; locationRemarks: label transliteration: "Kédougou, Ebarack, 2019.01.22, Mingou Paterne A.B. et al."; [Kédougou Ebarack 116 m, 12°37'49.2"N 012°52'19.4"W, 2019.01.22, Lithophyte, Mingou Paterne A.B. et al.]; verbatimCoordinates: 12°37'49.2"N 012°52'19.4"W; decimalLatitude: 12.6303; decimalLongitude: -12.872; georeferenceProtocol: GPS; **Identification:** identifiedBy: Mingou Paterne A.B.; dateIdentified: 2020; **Event:** samplingProtocol: sweeping; eventDate: 2019.01.22; habitat: Lithophyte; **Record Level:** language: fr; institutionCode: IFAN; collectionCode: Pteridophytes; ownerInstitutionCode: IFAN; basisOfRecord: PreservedSpecimen
- **Type status:** Other material. **Occurrence:** catalogNumber: IFAN63173; recordNumber: PABM0432; recordedBy: Mingou Paterne A.B. et al; occurrenceID: 8DD636F1-19EE-529F-8D31-762921123DD7; **Taxon:** taxonID: urn:lsid:ipni.org:names:77186660-1; scientificName: Selaginellasubcordata; scientificNameAuthorship: A.Braun ex Kuhn; **Location:** country: Senegal; stateProvince: Kédougou; locality: Southern part of the Dindefélo waterfall cliff, at 100 m high; verbatimElevation: 391 m; locationRemarks: label transliteration: "Kédougou, Dindefélo, 2020.10.06, Mingou Paterne A.B. et al."; [Kédougou Dindefélo 391 m, 12°21'54.3"N 012°19'30.1"W, 2020.10.06, Lithophyte, Mingou Paterne A.B. et al.]; verbatimCoordinates: 12°21'54.3"N 012°19'30.1"W; decimalLatitude: 12.3651; decimalLongitude: -12.325; georeferenceProtocol: GPS; **Identification:** identifiedBy: Mingou Paterne A.B.; dateIdentified: 2020; **Event:** samplingProtocol: climbing; eventDate: 2020.10.06; habitat: Lithophyte; **Record Level:** language: fr; institutionCode: IFAN; collectionCode: Pteridophytes; ownerInstitutionCode: IFAN; basisOfRecord: PreservedSpecimen
- **Type status:** Other material. **Occurrence:** catalogNumber: IFAN63174; recordNumber: PABM0434; recordedBy: Mingou Paterne A.B. et al; occurrenceID: D2B1F1E8-6CBD-5C76-9928-2FAE5CC93D83; **Taxon:** taxonID: urn:lsid:ipni.org:names:77186660-1; scientificName: Selaginellasubcordata; scientificNameAuthorship: A.Braun ex Kuhn; **Location:** country: Senegal; stateProvince: Kédougou; locality: Northern part of the Dindefélo waterfall cliff, at 90 m high; verbatimElevation: 385 m; locationRemarks: label transliteration: "Kédougou, Dindefélo, 2020.10.06, Mingou Paterne A.B. et al."; [Kédougou Dindefélo 385 m, 12°21'54.0"N 012°19'29.7"W, 2020.10.06, Lithophyte, Mingou Paterne A.B. et al.]; verbatimCoordinates: 12°21'54.0"N 012°19'29.7"W; decimalLatitude: 12.365; decimalLongitude: -12.3249; georeferenceProtocol: GPS; **Identification:** identifiedBy: Mingou Paterne A.B.; dateIdentified: 2020; **Event:** samplingProtocol: climbing; eventDate: 2020.10.06; habitat: Lithophyte; **Record Level:** language: fr; institutionCode: IFAN; collectionCode: Pteridophytes; ownerInstitutionCode: IFAN; basisOfRecord: PreservedSpecimen
- **Type status:** Other material. **Occurrence:** catalogNumber: IFAN63175; recordNumber: PABM0436; recordedBy: Mingou Paterne A.B. et al; occurrenceID: D11F7662-1B3F-5306-B827-1DF68E782258; **Taxon:** taxonID: urn:lsid:ipni.org:names:77186660-1; scientificName: Selaginellasubcordata; scientificNameAuthorship: A.Braun ex Kuhn; **Location:** country: Senegal; stateProvince: Kédougou; locality: Southern part of the Dindefélo waterfall cliff, at 60 m high; verbatimElevation: 369 m; locationRemarks: label transliteration: "Kédougou, Dindefélo, 2020.10.06, Mingou Paterne A.B. et al."; [Kédougou Dindefélo 369 m, 12°21'52.0"N 012°19'25.6"W, 2020.10.06, Lithophyte, Mingou Paterne A.B. et al.]; verbatimCoordinates: 12°21'52.0"N 012°19'25.6"W; decimalLatitude: 12.3644; decimalLongitude: -12.3238; georeferenceProtocol: GPS; **Identification:** identifiedBy: Mingou Paterne A.B.; dateIdentified: 2020; **Event:** samplingProtocol: climbing; eventDate: 2020.10.06; habitat: Lithophyte; **Record Level:** language: fr; institutionCode: IFAN; collectionCode: Pteridophytes; ownerInstitutionCode: IFAN; basisOfRecord: PreservedSpecimen
- **Type status:** Other material. **Occurrence:** catalogNumber: IFAN63176; recordNumber: PABM0437; recordedBy: Mingou Paterne A.B. et al; occurrenceID: 4D3A1D19-B26E-536A-A3AF-822D8B01137F; **Taxon:** taxonID: urn:lsid:ipni.org:names:77186660-1; scientificName: Selaginellasubcordata; scientificNameAuthorship: A.Braun ex Kuhn; **Location:** country: Senegal; stateProvince: Kédougou; locality: Southern part of the Dindefélo waterfall cliff, at 50 m high; verbatimElevation: 358 m; locationRemarks: label transliteration: "Kédougou, Dindefélo, 2020.10.06, Mingou Paterne A.B. et al."; [Kédougou Dindefélo 358 m, 12°21'53.4"N 012°19'27.7"W, 2020.10.06, Lithophyte, Mingou Paterne A.B. et al.]; verbatimCoordinates: 12°21'53.4"N 012°19'27.7"W; decimalLatitude: 12.3648; decimalLongitude: -12.3244; georeferenceProtocol: GPS; **Identification:** identifiedBy: Mingou Paterne A.B.; dateIdentified: 2020; **Event:** samplingProtocol: climbing; eventDate: 2020.10.06; habitat: Lithophyte; **Record Level:** language: fr; institutionCode: IFAN; collectionCode: Pteridophytes; ownerInstitutionCode: IFAN; basisOfRecord: PreservedSpecimen
- **Type status:** Other material. **Occurrence:** catalogNumber: IFAN63177; recordNumber: PABM0438; recordedBy: Mingou Paterne A.B. et al; occurrenceID: DCEC83FC-26AB-5D7F-93F2-D295984528A4; **Taxon:** taxonID: urn:lsid:ipni.org:names:77186660-1; scientificName: Selaginellasubcordata; scientificNameAuthorship: A.Braun ex Kuhn; **Location:** country: Senegal; stateProvince: Kédougou; locality: Northern part of the Dindefélo waterfall cliff, at 40 m high; verbatimElevation: 338 m; locationRemarks: label transliteration: "Kédougou, Dindefélo, 2020.10.06, Mingou Paterne A.B. et al."; [Kédougou Dindefélo 338 m, 12°21'53.8"N 012°19'27.1"W, 2020.10.06, Lithophyte, Mingou Paterne A.B. et al.]; verbatimCoordinates: 12°21'53.8"N 012°19'27.1"W; georeferenceProtocol: GPS; **Identification:** identifiedBy: Mingou Paterne A.B.; dateIdentified: 2020; **Event:** samplingProtocol: climbing; eventDate: 2020.10.06; habitat: Lithophyte; **Record Level:** language: fr; institutionCode: IFAN; collectionCode: Pteridophytes; ownerInstitutionCode: IFAN; basisOfRecord: PreservedSpecimen
- **Type status:** Other material. **Occurrence:** catalogNumber: IFAN63178; recordNumber: PABM0449; recordedBy: Mingou Paterne A.B. et al; occurrenceID: 8106D589-4161-59B4-B641-A5845F61DAFD; **Taxon:** taxonID: urn:lsid:ipni.org:names:77186660-1; scientificName: Selaginellasubcordata; scientificNameAuthorship: A.Braun ex Kuhn; **Location:** country: Senegal; stateProvince: Kédougou; locality: Southern part of the Dindefélo waterfall cliff, base of the cliff of the Dindefélo waterfall, at 1 m high; verbatimElevation: 291 m; locationRemarks: label transliteration: "Kédougou, Dindefélo, 2020.10.06, Mingou Paterne A.B. et al."; [Kédougou Dindefélo 291 m, 12°21'53.8"N 012°19'27.1"W, 2020.10.06, Lithophyte, Mingou Paterne A.B. et al.]; verbatimCoordinates: 12°21'53.8"N 012°19'27.1"W; georeferenceProtocol: GPS; **Identification:** identifiedBy: Mingou Paterne A.B.; dateIdentified: 2020; **Event:** samplingProtocol: climbing; eventDate: 2020.10.06; habitat: Lithophyte; **Record Level:** language: fr; institutionCode: IFAN; collectionCode: Pteridophytes; ownerInstitutionCode: IFAN; basisOfRecord: PreservedSpecimen
- **Type status:** Other material. **Occurrence:** catalogNumber: IFAN63179; recordNumber: PABM0453; recordedBy: Mingou Paterne A.B. et al; occurrenceID: 800DF1EB-3E3B-5AC3-86CF-0422737DE75E; **Taxon:** taxonID: urn:lsid:ipni.org:names:77186660-1; scientificName: Selaginellasubcordata; scientificNameAuthorship: A.Braun ex Kuhn; **Location:** country: Senegal; stateProvince: Kédougou; locality: Track leading to the Kounssy waterfall; verbatimElevation: 315 m; locationRemarks: label transliteration: "Kédougou, Kounssy, 2020.10.08, Mingou Paterne A.B. et al."; [Kédougou Kounssy 315 m, 12°25'39.2"N 012°04'49.3"W, 2020.10.08, Lithophyte, Mingou Paterne A.B. et al.]; verbatimCoordinates: 12°25'39.2"N 012°04'49.3"W; decimalLatitude: 12.4276; decimalLongitude: -12.0804; georeferenceProtocol: GPS; **Identification:** identifiedBy: Mingou Paterne A.B.; dateIdentified: 2020; **Event:** samplingProtocol: sweeping; eventDate: 2020.10.08; habitat: Lithophyte; **Record Level:** language: fr; institutionCode: IFAN; collectionCode: Pteridophytes; ownerInstitutionCode: IFAN; basisOfRecord: PreservedSpecimen
- **Type status:** Other material. **Occurrence:** catalogNumber: IFAN63180; recordNumber: PABM0536; recordedBy: Mingou Paterne A.B. et al; occurrenceID: 5A55DCCE-3356-58D3-8666-223DBD2B9F6A; **Taxon:** taxonID: urn:lsid:ipni.org:names:77186660-1; scientificName: Selaginellasubcordata; scientificNameAuthorship: A.Braun ex Kuhn; **Location:** country: Senegal; stateProvince: Kédougou; locality: Descending track from Lombel Cliff to Dimboli; verbatimElevation: 280 m; locationRemarks: label transliteration: "Kédougou, Lombel-Dimboli, 2021.11.28, Mingou Paterne A.B. et al."; [Kédougou Lombel-Dimboli 280 m, 12°26'48.7"N 011°59'46.9"W, 2021.11.28, Lithophyte, Mingou Paterne A.B. et al.]; verbatimCoordinates: 12°26'48.7"N 011°59'46.9"W; decimalLatitude: 12.4469; decimalLongitude: -11.9964; georeferenceProtocol: GPS; **Identification:** identifiedBy: Mingou Paterne A.B.; dateIdentified: 2021; **Event:** samplingProtocol: sweeping; eventDate: 2021.11.28; habitat: Lithophyte; **Record Level:** language: fr; institutionCode: IFAN; collectionCode: Pteridophytes; ownerInstitutionCode: IFAN; basisOfRecord: PreservedSpecimen
- **Type status:** Other material. **Occurrence:** catalogNumber: BR0000015471024; recordNumber: 841; recordedBy: Berhaut R.P.; occurrenceID: B06B3784-D86C-59B6-B1B0-65FC11C42E3E; **Taxon:** taxonID: urn:lsid:ipni.org:names:77186660-1; scientificName: Selaginellasubcordata; scientificNameAuthorship: A.Braun ex Kuhn; **Location:** country: Senegal; stateProvince: Tambacounda; locality: Niokolo-Koba; locationRemarks: label transliteration: "Tambacounda, Niokolo-Koba, 1951.04.00, Berhaut R.P."; [Tambacounda Niokolo-Koba, 1951.04.00, Berhaut R.P.]; georeferenceProtocol: Label; **Event:** samplingProtocol: none specified; eventDate: 1951.04.00; habitat: none specified; **Record Level:** language: fr; institutionCode: BR; collectionCode: Pteridophytes; ownerInstitutionCode: BR; basisOfRecord: PreservedSpecimen
- **Type status:** Other material. **Occurrence:** catalogNumber: BR0000015471284; recordNumber: 9145; recordedBy: Vanden Berghen C.; occurrenceID: 9DB4E85A-4DBB-58A2-BB98-CE504E3861F7; **Taxon:** taxonID: urn:lsid:ipni.org:names:77186660-1; scientificName: Selaginellasubcordata; scientificNameAuthorship: A.Braun ex Kuhn; **Location:** country: Senegal; stateProvince: Ziguinchor; locality: Basse-Casamance, Small damp embankment in the thicket, along the path, Dar Salam; verbatimElevation: 3 m; locationRemarks: label transliteration: "Ziguinchor, Basse-Casamance, 1990.10.25, Vanden Berghen C."; [Ziguinchor Basse-Casamance, 1990.10.25, Vanden Berghen C.]; georeferenceProtocol: Label; **Event:** samplingProtocol: none specified; eventDate: 1990.10.25; habitat: none specified; **Record Level:** language: fr; institutionCode: BR; collectionCode: Pteridophytes; ownerInstitutionCode: BR; basisOfRecord: PreservedSpecimen

#### Description

**Figure 3.**
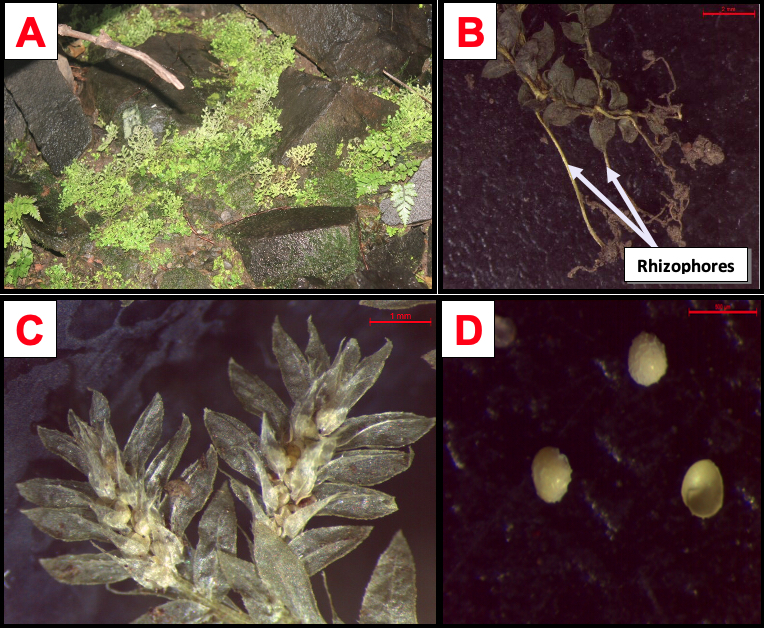
*Selaginellasubcordata* A.Braun ex Kuhn. **A** Plant in its environment; **B** Rhizophores located in the lower half of the stem; **C** Sporophylls dorsal and ventral; **D** Megaspores ochre yellow, finely rough.

Plant lithophytic (Fig. 3A). Rhizophores located in the lower half of the stem up to 2 cm long, glabrous, filiform, greenish-white making a right angle with the stem then curved downwards (Fig. 3B). Stem decumbent at the base, slightly ascending distally, up to 50 mm long, staminate, greenish, pale; branches glabrous, diverging by approximately 60°, bipinnate with oval outline. Leaves heteromorphic; lateral leaves spaced, unequal, about 2 mm long by 1.20 mm wide, apiculate, the upper half semi-ovate-oblong, cordate and surrounding the stem at the base, ciliate on the adaxial half, loosely serried towards the top (Fig. 3C); lower half semi-oblong, tightly rounded at the base. Median leaves distant on the main stem, imbricated on the branches, very small, ciliolate, toothed outline, oval in shape, acuminate, aristate at about 2/3 of the length of the blade. Strobile at the top of the main stem and branches approximately 2.5-3 mm long by 1-2.5 mm wide. Dimorphic sporophylls: dorsal sporophylls, oblong green, acuminate, shortly awned, irregularly serrulate with occasionally ciliate-toothed keel; ventral sporophylls hyaline, deltoid, acuminate, awned, ciliate; megaspores ochre yellow, finely rough on the distal surface (Fig. 3D).

#### Distribution

**Figure 4.**
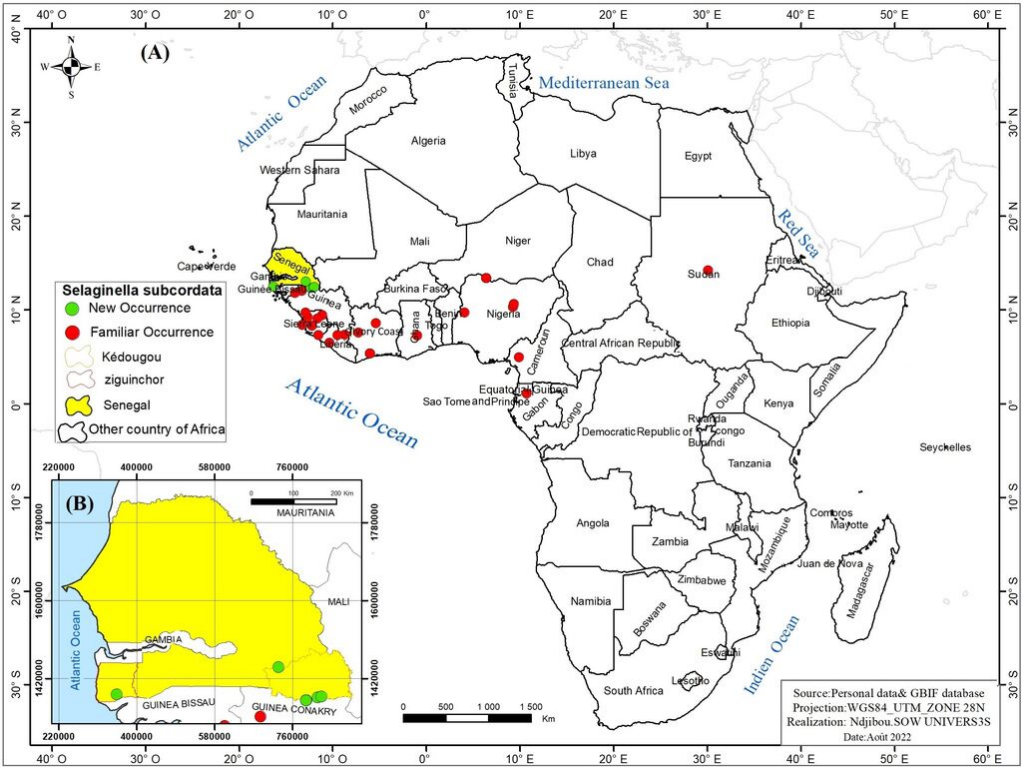
Distribution map *Selaginellasubcordata* A.Braun ex Kuhn in Africa **(A)** and Senegal **(B).**

Distribution in Senegal: Three Regions: Kédougou, Tambacounda and Ziguinchor (Fig. 4B).

Distribution in Africa: Cameroon, Ivory Coast, Gabon, Ghana, Guinea, Liberia, Nigeria, Sierra Leone and Sudan (GBIF.org 2022b) (Fig. 4A).

#### Ecology

*Selaginellasubcordata* is found as a lithophyte at altitudes between 116 and 483 m a.s.l., on either side of the Dindefélo Falls cliff, at 100 m, 60 m, 50 m and 1 m on the south side and 90 m, 40 m on the north side. The southern part of the cliff is humid and not very sunny, with a few species of trees, unlike the northern part which is less humid and very sunny. On the Dindefélo cliff, *S.subcordata* as well as *S.kraussiana* was found each time under plant cover in association with pteridophyte species such as *Aleutopterisfarinosa* (Forssk.) Fée, *Blotiellacurrorii* (Hook.) R.M. Tryon, *Dicranopterislinearis* (Burm.f.) Underw. and *Selaginellaversicolor* Spring, but also with byophytes and spermatophytes such as *Dombeyaquinqueseta* (Delie) Excell, *Mitragynainermis* (Willd.) K.Schum. and *Raphiasudanica* A.Chev. It was also found on the track going down the Dimboli cliff, but also on rocks not far from the Sédou stream bridge in Ebarack. In Ziguinchor, it was found on a small damp humid area, covered with bushes, along a path. In Tambacounda, the habitat of historical specimens from Niokolo-Koba Park, has not been mentioned.

#### Notes

*Selaginellasubcordata* was first collected in April 1951 by Berhaut J (BR0000020401276), then on 25 October 1990 by Vanden Berghen C. (BR0000015471284) and recently by Mingou et al. 2019, 2020 (See Senegalese materials examined above) and is reported for the first time for Senegal.

## Identification Keys

### Identification keys to the *Selaginella* species in Senegal

**Table:** 

1	Stems twining, glabrous, straw-coloured, with distant leaves	***Selaginellamyosurus***
–	Stems erect, arched or prostrate, never twining	2
2	Sporophylls uniform; lateral leaves serrate with two false veins, lower surface of the leaf light green, upper surface dark green	***Selaginellaversicolor***
–	Sporophylls dimorphic	3
3	Rhizophores on the upper surface of the stems, along its entire length; delicate leaves	***Selaginellakraussiana***
–	Rhizophores in lower half of stem	4
4	Lateral leaves with a subcordate upper base, covering the main stem	***Selaginellasubcordata***
–	Lateral leaves with rounded upper base, not covering the main stem, the lower base is more or less developed	***Selaginellatenerrima***

## Discussion

For the identification of Selaginellaceae species from Senegal, certain characters were essential such as the types of rhizome, sporophyll as well as the arrangement of the rhizophores. Therefore, these interspecific morphological differences in Selaginella are often indistinct or unclear and the group is known for problems with species identification (Korall and Kenrick 2002).

Pteridophytes inhabit a wide variety of substrates, climates and light regimes (Young and León 1989, Pausas and Sáez 2000, Aldasoro et al. 2004, Abotsi et al. 2020, Priambudi et al. 2022), both in habitats dominated by flowering plants and where few angiosperms can survive (Mir et al. 2014). From an ecological point of view, pteridophytes are much more confined to the south of Senegal which presents favourable conditions for their development thanks to its wetlands, its numerous waterfalls, rivers, gallery forests and its high forest areas (Mingou and Gueye 2017, Mingou et al. 2021, Mingou et al. 2023). In Senegal, the two new species records of *Selaginella* are both mainly lithophytic with a preference for humid, inaccessible places, which are important for their water requirement and for their good development. However, they do not have the same distribution presence in this country. *Selaginellakraussiana* is restricted to Kedougou, a region of Senegal at the level of the interstices of the cliff of the Dindefélo Waterfall, a habitat that it shares with *Selaginellasubcordata*. The latter is also found in the regions of Tambacounda and Ziguinchor on a small damp humid area, covered with bushes, along a path. These natural habitats in which we found them correspond well to their known ecology, particularly humid, shady, rocky places (Roux 2001, Roux 2003, Mangambu 2013, Médail and Quézel 2018).

*Selaginellakraussiana* and *Selaginellasubcordata* have been reported in other parts of the world, but they do not have the same geographical distribution in African or even at global level. *Selaginellakraussiana* is a subcosmopolitan species with distribution in Africa, Asia, America and Europe (GBIF.org 2022a). It is uncommon in West and Central Africa, while it is widely distributed in southern and eastern Africa. *S subcordata* is an African species that is mainly present in West Africa. The obtained results corroborate those of Aldasoro et al. (2004), who showed that the distribution areas of Pteridophytes are very variable, some covering large areas, others localised in small places. The present work also underlines the importance of floristic studies in the field and of herbarium samples, as demonstrated by several authors worldwide (e.g. Bartolucci et al. (2019), Park et al. (2023)).

## Supplementary Material

XML Treatment for Selaginella kraussiana

XML Treatment for Selaginella subcordata
